# Associations between nutritional status and cognitive impairment in older adults: results from the NHANES 2011–2014 cycles

**DOI:** 10.3389/fnut.2025.1571990

**Published:** 2025-07-03

**Authors:** Xuehe Zhang, Long Yang, Zhong Wang, Hanxiao Wang, Shiyu Nie, Chunxiu Zhao, Shengnan Kang, Chuan Zhang, Chuanliang Pan

**Affiliations:** ^1^Department of Intensive Care Unit, The Third People’s Hospital of Chengdu, Chengdu, China; ^2^Pediatric Cardiothoracic Surgery, First Affiliated Hospital of Xinjiang Medical University, Urumqi, China

**Keywords:** Controlling Nutritional Status, cognitive, older adults, NHANES, cross-sectional study

## Abstract

**Background:**

The Controlling Nutritional Status (CONUT) score is used to assess nutritional status. Given that malnutrition increases the risk of cognitive impairment, this study aimed to clarify the relationship between the CONUT score and cognitive impairment in older adults.

**Methods:**

Older adults aged 60–80 years in the National Health and Nutrition Examination Survey (NHANES) cycles were included in this study. Cognitive impairment was identified according to three different cognitive assessment methods. The degree of malnutrition was assessed by calculating the CONUT score and categorized into normal nutrition (CONUT 0–1) and malnutrition (CONUT 2–12) groups based on the score. Independent associations between the CONUT score and cognitive impairment were examined using logistic regression, while non-linear associations between the CONUT score and cognitive impairment were investigated using restricted cubic splines (RCS).

**Results:**

The mean participant age was 69.19 years, and approximately 1478 participants (53.6%) had cognitive impairment. According to the CONUT score, the incidence of cognitive impairment was significantly greater in the malnutrition group. According to the adjusted logistic regression analysis, the CONUT score was associated with cognitive impairment (OR: 1.133, 95% CI: 1.052, 1.221; *P* = 0.002). Cognitive impairment was also associated with the malnutrition group (OR: 1.259, 95% CI: 1.039, 1.524; *P* = 0.021). RCS analysis revealed L-shaped associations between the CONUT score and cognitive impairment (non-linear *p* < 0.001).

**Conclusion:**

Elevated CONUT scores are associated with an increased prevalence of cognitive impairment in older adults, potentially aiding in identifying individuals at risk of cognitive decline.

## 1 Introduction

As the world’s population ages, cognitive impairment has become more prevalent (1). As a growing public health problem, cognitive impairment now poses a significant challenge to health care systems. Studies have shown that even mild cognitive impairment results in dementia in 18.4%–32.3% of those individuals (2, 3). Research suggests that cognitive decline is associated with functional decline among older adults (4) and that cognitive impairment may increase the risk of disability (5). While acetylcholinesterase inhibitors and NMDA receptor antagonists have been approved for the treatment of Alzheimer’s disease dementia, their effects on age-related cognitive decline remain limited and primarily focus on symptomatic relief. Current disease research highlights the urgent need to shift focus toward preventive strategies targeting modifiable risk factors through non-pharmacological interventions (6). It has been shown that a healthy lifestyle can slow cognitive decline (7). Cognitive decline is the result of multiple factors, and the combination of advanced age and other risk factors is associated with multifaceted interactions that occur during the process of cognitive decline (8). Older adults face a variety of health problems, among which malnutrition is very common (9). Nutrition is a relevant healthy lifestyle factor that slows cognitive decline. Studies show that a progressive increase is observed in nutritional risk among older adults as opposed to younger adults (10). Aging-related changes in metabolism and body composition can lead to decreased appetite and food intake, which further increases fat and muscle tissue loss and causes malnutrition (11, 12). Studies have shown that cognitive decline and worsening nutritional status are closely related and that the majority of patients with cognitive impairment are at risk for malnutrition (13, 14). Multiple screening tools have been developed to identify nutritional risks, but no gold standard is available for the assessment of malnutrition. Nutritional Risk Screening 2002 (NRS 2002) considers nutritional assessment, disease severity, and the patient’s age and is recommended for the evaluation of hospitalized patients (15). Some older adults have difficulty completing structured questionnaires or accurately describing weight loss in the absence of caregivers. The Mini Nutritional Assessment Short Form (MNA-SF) has been more widely used in older adults, and in addition to nutritional risk, the MNA score has been associated with functional decline (16). However, this scale may not be sufficiently accurate for patients with unrecognized dementia or depression. Despite the multitude of tools available for nutritional assessment, these tools still have some limitations, and subjective assessments are inevitable. Both subjective and objective data should be collected for a comprehensive nutritional assessment.

Objective nutritional assessment tools quantify malnutrition risk by integrating biochemical markers (e.g., serum albumin, lymphocyte count) and anthropometric parameters (e.g., BMI, weight loss). Commonly used tools include the Controlling Nutritional Status (CONUT), Geriatric Nutritional Risk Index (GNRI), Prognostic Nutritional Index (PNI), and Nutritional Risk Index (NRI). Studies by Wang et al. (17) utilizing NHANES data demonstrated a significant correlation between GNRI levels and cognitive function in older adults. Similarly, Lee et al. (18) reported that GNRI independently predicts post-stroke cognitive impairment (PSCI) risk. Wang et al. (19) found that reduced PNI levels were associated with PSCI incidence, though no intergroup differences in GNRI were observed among stroke survivors. The utility of the CONUT score in cognitive-impaired populations remains underexplored. Previous studies linked CONUT to prognoses in colorectal cancer, heart failure, and hypertension patients (20–22). Liu et al. (23) identified high CONUT scores as the strongest predictors of in-hospital mortality among hospitalized older adults compared to four other nutritional assessment tools. The CONUT score is assessed via objective laboratory indices, which reduces inaccuracies in nutritional assessment due to patient recall bias. Therefore, the purpose of this study was to clarify the relationship between the CONUT score and cognitive function in an older population.

## 2 Materials and methods

### 2.1 Study subjects and sample sources

This cross-sectional study utilized data from the National Health and Nutrition Examination Survey (NHANES) 2011–2014 cycles. NHANES, designed by the National Center for Health Statistics (NCHS) under the Centers for Disease Control and Prevention (CDC), employs a stratified, multistage probability sampling design to assess the health status of non-institutionalized United States civilians. Since 1999, the survey has been conducted biennially with comprehensive physical examinations. During the 2011–2014 cycles, NHANES implemented cognitive function assessments specifically targeting older adults aged 60–80 years. From 3472 participants who completed cognitive questionnaires across both cycles, we excluded those with missing CONUT scores. The original NHANES protocol received ethical approval from the NCHS Research Ethics Review Board (Protocol #2011–17 and Continuation of Protocol #2011–17), with all participants providing written informed consent. As this investigation constitutes a secondary analysis of publicly available de-identified data, no additional institutional review board approval was required.

### 2.2 Data collection

This study utilized data from the NHANES database, where trained professionals collected demographic characteristics, laboratory measurements, and questionnaire responses through standardized protocols. All data are publicly accessible via the NHANES website^1^. Demographic variables included age, gender, race, marital status, annual household income, and educational attainment. Laboratory analyses encompassed lymphocyte/neutrophil counts, total cholesterol (TC), and other biochemical parameters. Dietary nutrient intake – including energy, protein, total saturated fatty acid, carbohydrate, and fiber – was assessed through 24-h dietary recall interviews. Cardiovascular disease (CVD) status was determined by self-reported physician diagnosis. Hypertension was defined as either (1) self-reported antihypertensive medication use, (2) systolic/diastolic blood pressure ≥ 140/90 mmHg (≥ 130/80 mmHg for diabetic patients), or (3) physician-diagnosed hypertension. Diabetes mellitus diagnosis required meeting at least one criterion: hemoglobin A1c (HbA1c) ≥ 6.5%, fasting glucose ≥ 126 mg/dL, random glucose ≥ 200 mg/dL, 2-h oral glucose tolerance test (OGTT) ≥ 200 mg/dL, or current use of glucose-lowering agents. NHANES classified medications by analyzing brand names or active ingredients and reclassified them into therapeutic categories. We specifically extracted medications potentially influencing CONUT scores, including lipid-lowering agents, corticosteroids, and immunosuppressants.

### 2.3 Diagnostic assessment of cognitive impairment

Three distinct methodologies were employed for the cognitive assessment of older adults aged 60 years or older within the NHANES. These methodologies included the Consortium to Establish a Registry for Alzheimer’s disease (CERAD) Word Learning (WL) subtest (24), the Animal Fluency Test (AFT) (25), and the Digit Symbol Substitution Test (DSST) (26). These tests comprehensively evaluate various cognitive functions, including working memory, language skills, processing speed, and executive function.

The CERAD test comprises three successive learning trials (CERAD-WL) and a delayed recall phase (CERAD-DR), which collectively measure the immediate and delayed acquisition of novel linguistic information. In the CERAD-WL task, participants vocalized 10 unrelated words individually, with the order shuffled across the three learning trials. Approximately 10 min after the initiation of the CERAD-WL task, the CERAD-DR task commenced, during which participants were asked to recall as many words as possible.

The AFT, a widely employed tool for evaluating language fluency in clinical contexts, taps into memory and language capabilities (including facets such as naming, comprehension, and semantic knowledge). Within a 1-min timeframe, participants were asked to name as many different types of animals as they could, earning one point for each animal that was correctly named.

Serving as a component of the Wechsler Adult Intelligence Scale (WAIS-III), the DSST focuses on processing speed, sustained attention, and working memory. The participants received a test sheet containing 9 numbers alongside corresponding symbols and a subsequent set of 133 numbers beneath the key. Their challenge was to quickly pair the accurate symbol with each number within a 2-min window.

The cutoff point for identifying cognitive impairment was determined by selecting the CERAD-WL, CERAD-DR, AFT, and DSST scores in the lowest quartile. This approach aligns with established methods in the literature for diagnosing cognitive impairment (26). Individuals with scores equal to or below this cutoff point were classified as having cognitive impairment. In this particular study, the cutoff values were as follows: 17 points for the CERAD-WL subtest, 5 points for the CERAD-DR subtest, 14 points for the AFT, and 33 points for the DSST.

### 2.4 Nutritional assessment

This study employed the CONUT score to evaluate participants’ nutritional status. The CONUT score was calculated based on serum TC levels, lymphocyte counts, and serum albumin concentrations (Supplementary Table 1). Scores range from 0 to 12 according to the predefined algorithm, with higher values indicating poorer nutritional status. Given the limited number of participants classified as moderate or severe malnutrition (CONUT ≥ 5), we dichotomized participants into two groups for analytical purposes: normal nutrition (CONUT 0–1) and malnutrition (CONUT 2–12).

### 2.5 Statistical analysis

All statistical analyses were conducted using R software (version 4.3.1) with appropriate weighting procedures through the “survey” package, in accordance with NHANES analytical guidelines. Participants were stratified into normal nutrition (CONUT 0–1) and malnutrition (CONUT 2–12) groups for baseline characteristic comparisons. Categorical variables were compared using Rao-Scott’s χ^2^ tests and reported as frequencies with weighted percentages, while continuous variables were analyzed via weighted *t*-tests and presented as weighted means ± standard deviations. The associations between nutritional status (both categorical groups and continuous CONUT scores) and cognitive function were evaluated using univariable and multivariable logistic regression models. Dose-response relationships between CONUT scores and cognitive impairment were visualized through restricted cubic spline (RCS) analysis with three knots. Missing covariates with < 20% missingness were retained in analyses, and missing values were imputed using multiple imputation by chained equations (MICE package in R). A two-sided *P*-value < 0.05 defined statistical significance for all analyses.

## 3 Results

### 3.1 Baseline characteristics of the participants

**FIGURE 1:**
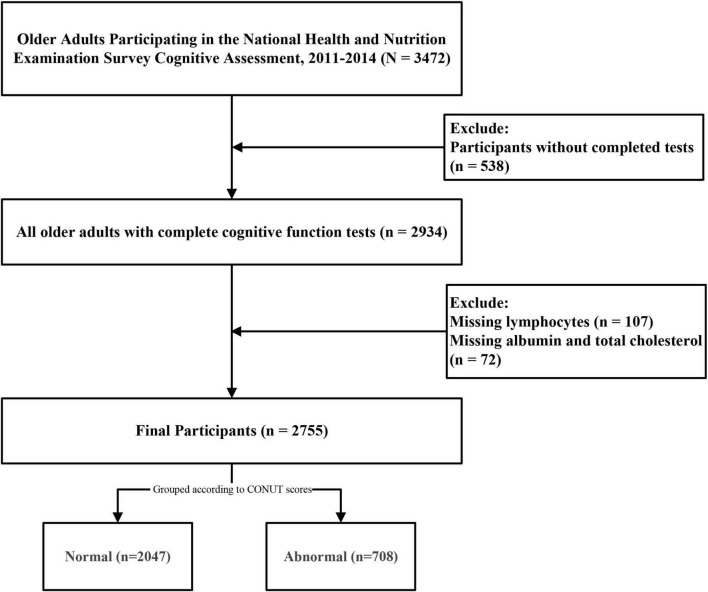
Flowchart of the study design.

**FIGURE 2:**
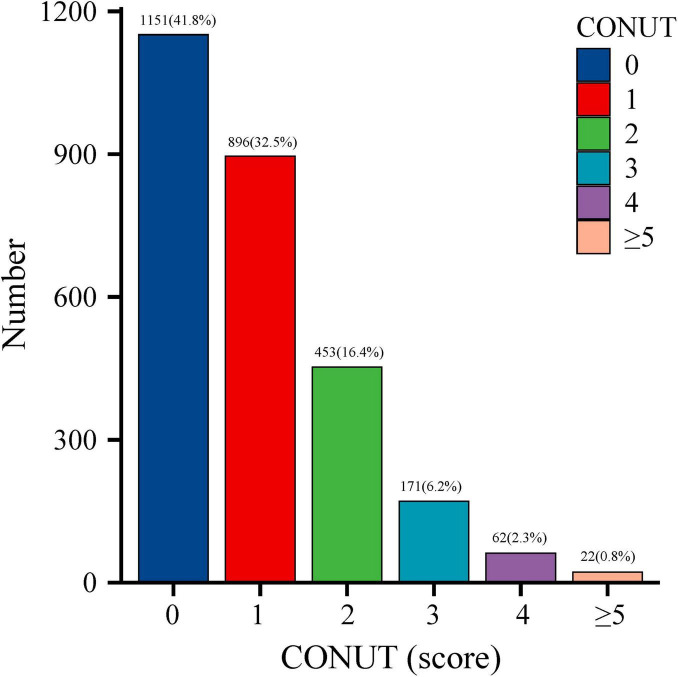
Distribution of Controlling Nutritional Status (CONUT) scores in the older adult population.

The initial study cohort comprised 3,472 older adults who completed cognitive questionnaires. After excluding 538 participants with incomplete cognitive assessments, 107 lacking lymphocyte count data, and 72 with missing albumin or TC measurements, the final analytical sample included 2,755 participants aged 60–80 years (Figure 1). Applying sample weights, this cohort represents approximately 50,801,593 older United States adults. The CONUT score distribution histogram (Figure 2) revealed that 74.3% of participants maintained good nutritional control (scores 0–1), while 24.9% exhibited moderate malnutrition (scores 2–4), and 0.8% demonstrated severe malnutrition (scores ≥ 5). Table 1 presents baseline characteristics stratified by nutritional status. The weighted mean age was 69.19 ± 0.19 years, with 1,352 participants (49.1%) being male. Cognitive impairment was identified in 1,478 participants (53.6%), showing a higher prevalence in the malnutrition group (*P* < 0.05). The malnutrition group also demonstrated significantly greater proportions of older individuals, males, CVD patients, and diabetics (all *P* < 0.05). No significant between-group differences (*P* > 0.05) were observed for body mass index (BMI), race, marital status, annual household income, educational attainment, hypertension status, dietary nutrient intake, or neutrophil counts. Medication analysis revealed higher usage rates of immunosuppressants and lipid-lowering agents in the malnutrition group (*P* < 0.05).

**TABLE 1:** Baseline characteristics of the study population by CONUT grouping.

Characteristics	Total	Normal	Malnutrition	*P*-value
	*N* = 2755	*N* = 2047	*N* = 708	
**Cognitive disorder**				
Cognitive	1478 (53.6)	1035 (38.0)	443 (52.4)	<0.001
CERAD-WL	767 (27.8)	523 (19.3)	244 (28.5)	<0.001
CERAD-DR	698 (25.3)	471 (19.7)	227 (26.2)	<0.001
AFT	817 (29.7)	576 (19.4)	241 (26.9)	0.002
DSST	700 (25.4)	492 (13.0)	208 (19.1)	<0.001
**Demographic**				
Age, year	69.19 ± 0.19	68.61 ± 0.20	70.93 ± 0.28	<0.001
BMI, kg/m2	29.01 ± 0.20	29.03 ± 0.22	28.93 ± 0.35	0.795
**Gender**				<0.001
Male	1352 (49.1)	873 (40.1)	479 (64.6)	
Female	1403 (50.9)	1174 (59.9)	229 (35.4)	
Race				0.113
Non-Hispanic White	1348 (48.9)	961 (79.6)	387 (82.7)	
Non-Hispanic Black	624 (22.6)	472 (8.0)	152 (7.6)	
Mexican American	244 (8.9)	186 (3.4)	58 (3.1)	
Other race	539 (19.6)	428 (9.0)	111 (6.6)	
Marital status				0.488
With-partner	1599 (58)	1175 (64.5)	424 (66.3)	
No-partner	1156 (42)	872 (35.5)	284 (33.7)	
Annual household income				0.429
More than 20,000	2183 (79.2)	1604 (86.8)	579 (88.1)	
Less than 20,000	572 (20.8)	443 (13.2)	129 (11.9)	
Education				0.464
High school or above	2443 (88.7)	1815 (94.3)	628 (94.7)	
Less than high school	312 (11.3)	232 (5.7)	80 (5.3)	
Cardiovascular disease	610 (22.1)	366 (18.3)	244 (33.9)	<0.001
Diabetes	1196 (43.4)	841 (35.9)	355 (44.8)	<0.001
Hypertension	1934 (70.2)	1418 (65.9)	516 (67.1)	0.672
**Dietary nutrient intake**				
Total saturated fatty acid	23.62 ± 0.33	23.34 ± 0.40	24.47 ± 0.76	0.222
Energy intake	1894.88 ± 20.33	1881.14 ± 24.25	1936.53 ± 36.25	0.214
Protein intake	73.54 ± 0.82	72.94 ± 0.99	75.34 ± 1.65	0.244
Carbohydrate intake	226.38 ± 3.14	224.65 ± 3.66	231.62 ± 5.71	0.305
Fiber intake	17.21 ± 0.37	17.07 ± 0.39	17.64 ± 0.58	0.307
Cholesterol intake	263.18 ± 5.19	258.19 ± 6.35	278.31 ± 13.35	0.216
**Laboratory examination**				
Lymphocyte, 10^9/L	1.91 ± 0.03	2.07 ± 0.03	1.42 ± 0.04	<0.001
Neutrophil, 10^9/L	4.22 ± 0.05	4.19 ± 0.06	4.29 ± 0.08	0.328
Albumin, g/L	42.11 ± 0.09	42.37 ± 0.09	41.31 ± 0.16	<0.001
TG, mg/dL	158.40 ± 3.36	178.37 ± 4.06	97.98 ± 5.12	<0.001
TC, mg/dL	193.00 ± 1.21	204.16 ± 1.09	159.21 ± 2.40	<0.001
**Drug utilization**				
Immunosuppressant	32 (0.9)	16 (0.7)	16 (1.5)	0.027
Antihyperlipidemic	1243 (46.4)	825 (41.9)	418 (60.0)	<0.001
Steroids	283 (11.5)	211 (11.7)	72 (10.7)	0.621

BMI, body mass index; TG, triglyceride; TC, total cholesterol.

### 3.2 Logistic regression analysis of the CONUT score and cognitive impairment in older adults

Logistic regression analysis demonstrated a significant positive association between CONUT scores and cognitive impairment (Table 2). In the crude model, elevated CONUT scores were linked to increased odds of cognitive impairment (OR: 1.345 [95% CI, 1.254–1.442]). Similarly, direct correlations were observed between CONUT scores and CERAD-WL, CERAD-DR, AFT, and DSST performance. Model 1 adjusted for age, BMI, and gender; Model 2 further incorporated comorbidities (diabetes mellitus, cardiovascular disease, hypertension); Model 3 additionally adjusted for medications influencing CONUT components. After comprehensive adjustment for confounders, CONUT scores retained a statistically significant association with cognitive impairment (OR: 1.133 [95% CI, 1.052–1.221]), alongside AFT (OR: 1.180 [95% CI, 1.036–1.344]) and DSST (OR: 1.126 [95% CI, 1.017–1.247]). Analyses grouped by nutritional level by CONUT showed that cognitive impairment was associated with malnutrition (OR: 1.783 [95% CI, 1.505–2.111]), which remained associated with malnutrition after adjustment for covariates (OR: 1.259 [95% CI, 1.039–1.524]).

**TABLE 2:** Logistic regression between CONUT and cognitive impairment and composition.

Characteristics	Crude model		Model 1		Model 2		Model 3	
	OR 95% CI	*P*-value	OR 95% CI	*P*-value	OR 95% CI	*P*-value	OR 95% CI	*P*-value
**CONUT score**								
Cognitive	1.345 (1.254, 1.442)	<0.001	1.160 (1.080, 1.246)	<0.001	1.128 (1.045, 1.217)	0.003	1.133 (1.052, 1.221)	0.002
CERAD-WL	1.290 (1.196, 1.391)	<0.001	1.112 (1.014, 1.220)	0.025	1.100 (1.004, 1.205)	0.041	1.085 (0.975, 1.209)	0.129
CERAD-DR	1.183 (1.119, 1.252)	<0.001	0.990 (0.926, 1.059)	0.772	0.967 (0.907, 1.031)	0.289	0.958 (0.892, 1.028)	0.22
AFT	1.268 (1.122, 1.432)	<0.001	1.193 (1.052, 1.353)	0.007	1.180 (1.035, 1.345)	0.016	1.180 (1.036, 1.346)	0.015
DSST	1.274 (1.165, 1.392)	<0.001	1.150 (1.043, 1.268)	0.007	1.115 (1.008, 1.232)	0.035	1.126 (1.017, 1.247)	0.025
**CONUT group**								
Normal	Reference		Reference		Reference		Reference	
Cognitive	1.783 (1.505, 2.111)	<0.001	1.303 (1.082, 1.568)	0.007	1.255 (1.035, 1.522)	0.023	1.259 (1.039, 1.524)	0.021
CERAD-WL	1.677 (1.419, 1.981)	<0.001	1.198 (0.987, 1.454)	0.067	1.189 (0.974, 1.451)	0.087	1.149 (0.924, 1.429)	0.199
CERAD-DR	1.454 (1.195, 1.770)	<0.001	0.991 (0.805, 1.219)	0.929	0.957 (0.787, 1.165)	0.651	0.937 (0.768, 1.144)	0.505
AFT	1.504 (1.156, 1.957)	0.003	1.295 (0.989, 1.697)	0.059	1.286 (0.972, 1.703)	0.076	1.272 (0.967, 1.674)	0.082
DSST	1.593 (1.264, 2.009)	<0.001	1.263 (0.974, 1.637)	0.076	1.134 (0.867, 1.483)	0.344	1.162 (0.887, 1.523)	0.261

The crude model adjusted for none. Model 1 was adjusted for age, BMI, and gender. Model 2 was adjusted for age, BMI, gender, diabetes mellitus, cardiovascular disease, and hypertension. Model 3 was adjusted for age, BMI, gender, diabetes mellitus, cardiovascular disease, hypertension, and medications affecting the CONUT component.

### 3.3 Dose-response relationships between the CONUT score and cognitive impairment and its components

**FIGURE 3:**
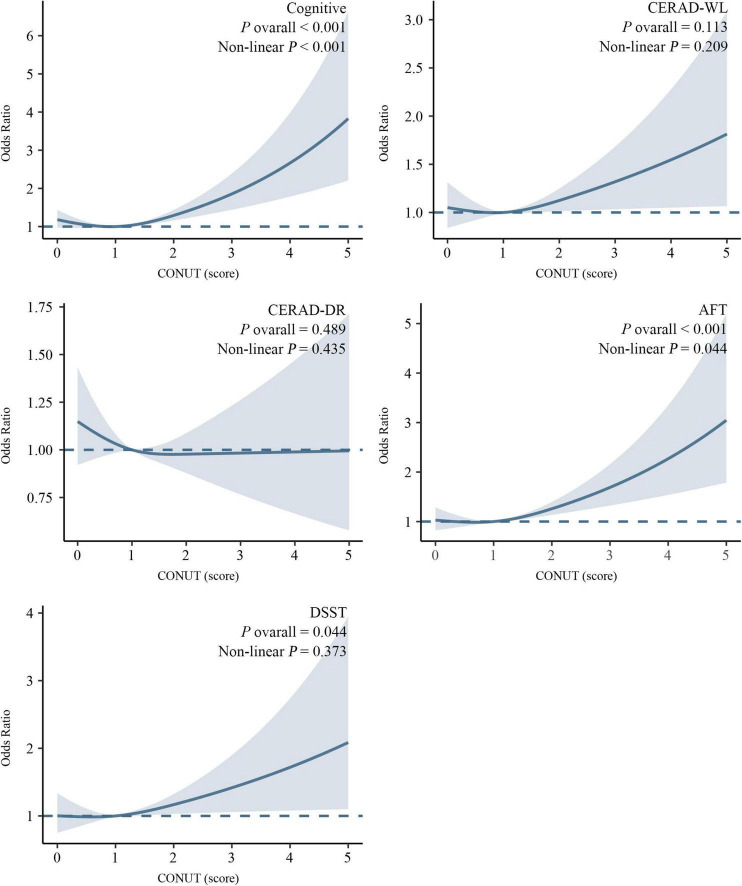
Association between the CONUT score and cognitive impairment revealed by restricted cubic spline (RCS) analysis. Adjusted for age, BMI, gender, diabetes mellitus, cardiovascular disease, hypertension, and medications affecting the CONUT component.

Restricted cubic spline analyses revealed a significant L-shaped association between CONUT scores and cognitive impairment (Figure 3). Specifically, an increasing risk of cognitive impairment was observed with elevated CONUT scores, demonstrating a statistically significant non-linear relationship (P for non-linearity < 0.001). A similar L-shaped pattern was identified between CONUT and AFT (P for non-linearity = 0.044). Moreover, a linear relationship was found between CONUT and DSST (P for non-linearity = 0.373). In contrast, no significant associations were detected between CONUT and CERAD-WL or CERAD-DR (*P* overall > 0.05).

## 4 Discussion

Our NHANES-based study provides robust epidemiological evidence for the association between nutritional status (assessed via CONUT score) and cognitive impairment in older adults. The findings demonstrate that elevated CONUT scores maintain an independent and dose-dependent association with cognitive impairment, even after rigorous adjustment for clinical baseline characteristics and pharmacological confounders.

With increasing age, the structure of the human brain changes. Age-related diseases often lead to cognitive decline in older adult individuals. Pathological changes in the brain appear to precede clinical manifestations, which provides a considerable amount of time for the effective implementation of preventive strategies. A wide range of factors, such as dysphagia, speech disorders, depression and medication side effects, affect nutritional status. Clinical consequences of malnutrition include functional decline, impairment in performing daily activities, and increased mortality (27, 28). Older adults with cognitive impairment also have higher rates of malnutrition, and weight loss and dietary changes occur prior to impairment (13, 29). Given the specific energy metabolism pattern of the brain, cognitive decline depends not only on the deterioration of brain structure and function, but also on adequate nutrient intake and nutritional status, which play important roles in maintaining cognitive function (28). There is a close relationship between nutrition and the brain, which needs adequate nutrition to maintain cognitive function (28).

The role of nutritional interventions in mitigating cognitive decline has been substantiated through diverse dietary patterns and nutrient supplementation strategies (30). Malnutrition disrupts cognitive function, promotes muscle and bone loss, causes immunosuppression and increases mortality. Evidence indicates that unhealthy dietary habits and poor nutritional status are associated with the progression of cognitive impairment risk (31). Among post-stroke patients with cognitive impairment, malnutrition is prevalent, with nutritional risk demonstrating prognostic relevance to cognitive outcomes (32). Older adults constitute a high-risk population for malnutrition. Community-dwelling older adults in China exhibiting nutritional risk show elevated susceptibility to cognitive impairment (33). Findings from the Singapore Longitudinal Ageing Studies reveal that individuals aged ≥ 55 years with increased nutritional risk exhibit higher vulnerability to cognitive impairment (34). Similarly, malnutrition in Greek older adults demonstrates significant associations with both cognitive decline and depressive symptoms (35).

Therefore, nutritional interventions are critical to prevent cognitive decline. Systematic screening for malnutrition risk to determine the presence and severity of malnutrition contributes to an improved prognosis as well as to the successful management of malnutrition-related complications. The ultimate manifestation of malnutrition results from multifactorial determinants, yet standardized screening instruments remain lacking for identifying these contributing factors. Concurrently, substantial heterogeneity across studies due to the multiplicity of screening tools complicates inter-study comparability (15). Given these challenges, distinct screening instruments are required for nutritional risk assessment in diverse patient populations, with particular considerations for individuals with cognitive impairment. The MNA-SF, while widely utilized in geriatric populations, relies on subjective self-reporting and fails to evaluate feeding abnormalities secondary to cognitive or psychiatric symptoms, rendering it suboptimal for assessing cognitively impaired individuals. Similarly, the Simplified Nutritional Appetite Questionnaire (SNAQ) necessitates patient-reported information, potentially introducing bias when relying on caregiver proxies. The NRS 2002 and Malnutrition Universal Screening Tool (MUST) share analogous limitations, being primarily designed for acute nutritional risk evaluation rather than chronic cumulative nutritional deficits prevalent in cognitive impairment populations. The study showed that the agreement of malnutrition classification between the MNA-SF and Global Leadership Initiative on Malnutrition (GLIM) criteria was low among community-dwelling older adults, with less than 25% of participants receiving consistent classification results (36). Objective laboratory biomarkers reflecting nutritional alterations may prove more applicable. However, even the objective nutritional assessment tools exhibit specific limitations in older adult populations. Anthropometric parameters are not routinely monitored in older adults, and abrupt weight changes may be confounded by edema, dehydration, or acute illnesses rather than true nutritional status. Standardized measurements of weight and height are often challenging to obtain. For individuals with cognitive impairment, weight-based metrics in the NRI are more susceptible to assessment bias. Among these tools, the CONUT score shares components with the PNI-except for TC levels-but differs in methodology. Unlike the PNI, which requires continuous numerical calculations, the CONUT score employs categorical thresholds, making it more practical for rapid clinical classification. Notably, components of the CONUT score may be influenced by medications. To address this potential confounding, our study explicitly adjusted for medication use in the analytical framework. To our knowledge, research investigating CONUT in relation to cognitive impairment remains scarce compared to other nutritional scores or questionnaires. Previous studies in schizophrenia patients comparing three nutritional assessments, including CONUT, demonstrated non-significant correlations between CONUT and cognitive impairment (37). Our findings reveal a significantly elevated prevalence of cognitive impairment in populations with CONUT-quantified malnutrition. Further analysis establishes that elevated CONUT scores independently associate with cognitive impairment risk in older adults, aligning with prior studies linking nutritional deficiencies to cognitive decline.

Within the CONUT components, hypoalbuminemia not only indicates malnutrition but also reflects systemic inflammation (38). It has been found that people who experience cognitive impairment have lower serum albumin levels. Kim et al further clarified the relationship between low serum albumin levels and neurodegenerative diseases (39). Wang et al. (40) showed that low serum albumin levels increased the risk of cognitive impairment even in subjects with normal cognitive function. A study by Llewellyn et al. (41) also suggested that low serum albumin levels are associated with cognitive impairment in individuals older than 65 years. The aging process is accompanied by a decline in function and the accumulation of oxidative damage; this is paralleled by an increase in the brain’s susceptibility to damage and deterioration in structure and function, which leads to regression in cognitive behavior (42). Owing to the height of lipid content and oxidative metabolism in neurons, the brain is susceptible to oxidative stress, especially during aging and age-related cognitive impairment (43). Serum albumin, the primary antioxidant in plasma, plays a key role in oxidative damage. As part of the immune response, lymphocytes play an important role. Inadequate nutritional intake may lead to decreased lymphocyte counts and leads to functional alterations in cellular and humoral responses (44, 45). Some studies have reported a decrease in lymphocyte count in progressive malnutrition (46, 47). Low lymphocyte counts have also been reported in animals with cognitive impairment (48). Cholesterol levels decrease with age due to reasons that may be related to chronic disease, poor health, and metabolic and hormonal changes. Solomon et al. (49) showed that the relationship between TC and cognitive impairment varies by age. The decline in TC levels in later life increases the risk of cognitive impairment and is associated with the progressive course of cognitive decline. Aine et al. (50) found that participants with high TC levels had more accurate verbal memory and that the two were positively correlated. Thus, some researchers believe that low TC levels in older adults may impair brain structure/function and cognition.

Previous studies indicate that nutritional interventions yield greater benefits in specific populations with baseline cognitive impairment, particularly demonstrating improvements in attention, processing speed, and memory among older adults (51). The CERAD cognitive battery includes tasks assessing episodic memory capacity and facilitates early detection of cognitive decline. The AFT evaluates semantic fluency, while the DSST exhibits high sensitivity to cognitive changes, particularly processing speed. Our study revealed differential associations between CONUT scores and domain-specific cognitive functions. A stronger association was observed between CONUT and AFT performance, suggesting malnutrition may particularly affect cognitive processes dependent on prefrontal and temporal regions. However, the ceiling effect in certain CERAD memory tasks may obscure nutritional influences on cognitive performance, necessitating cautious interpretation.

The relationship between malnutrition and cognitive impairment is well established, and while many screening tools for malnutrition exist, most of them require detailed training by health care professionals and that participants have normal cognitive function. The CONUT score is simply calculated via objective indicators. This score is not only used for malnutrition grading, but also closely related to the occurrence of cognitive impairment in patients. The CONUT score considers a patient’s immune status, as well as protein and lipid storage, and reductions in each of these elements are associated with a poorer nutritional status. Our study suggests that the CONUT score can serve as a tool for identifying cognitive impairment in older adults, with demonstrated significant associations between CONUT and the risk of cognitive impairment in aging populations. However, this study has several limitations. The cross-sectional design precludes definitive conclusions regarding the temporal sequence and causal relationship between CONUT scores and cognitive impairment. Notably, cognitive impairment may itself contribute to malnutrition through mechanisms such as reduced dietary intake or impaired self-care, yet the cross-sectional framework cannot establish the directionality of this association. Although adjustments were made for demographic characteristics, comorbidities, and medication use, residual confounding from unmeasured variables (e.g., inflammatory biomarkers, genetic predisposition) may persist. The identification of cognitive impairment relied on psychometric test thresholds rather than clinically validated diagnostic criteria. This approach risks misclassifying individuals with normal cognitive function or failing to detect mild cognitive impairment, thereby limiting the external validity of findings. Additionally, the limited proportion of participants with severe malnutrition (CONUT score ≥ 5) restricted robust evaluation of the relationship between extreme nutritional deficits and cognitive outcomes.

## 5 Conclusion

This study found a greater prevalence of cognitive impairment among the participants in the malnutrition group. Even after adjusting for potential confounders, cognitive impairment was independently associated with the CONUT score. Given the important role of malnutrition in cognitive impairment, this study’s findings suggest that the CONUT score can help in the identification of older adult individuals with cognitive impairment.

## Data Availability

Publicly available datasets were analyzed in this study. This data can be found here: https://www.cdc.gov/nchs/nhanes/.

## Footnotes

1
https://wwwn.cdc.gov/nchs/nhanes/
