# Supplementary material for: Associations between nutritional status and cognitive impairment in older adults: results from the NHANES 2011–2014 cycles
Source: Front Nutr. 2025 Jul 3;12:1571990. doi: 10.3389/fnut.2025.1571990 (PMC12267017; doi:10.3389/fnut.2025.1571990)
Supplement: Supplementary file 1 [file Table_1.docx]

**Supplementary Table 1**

| Characteristics | Grade | | | |
| --- | --- | --- | --- | --- |
| Serum albumin (g/L) | ≥35 | 30-34.9 | 25-29.9 | <25 |
| score | 0 | 2 | 4 | 6 |
| Total cholesterol (mg/dl) | >180 | 140-180 | 100-139 | <100 |
| score | 0 | 1 | 2 | 3 |
| Lymphocyte count (10^9/L) | ≥1.6 | 1.2-1.59 | 0.8-1.19 | <0.8 |
| score | 0 | 1 | 2 | 3 |
| CONUT | 0-1 | 2-4 | 5-8 | 9-12 |
|  | normal | mild | moderate | severe |
