# Supplementary material for: Associations between nutritional status and cognitive impairment in older adults: results from the NHANES 2011–2014 cycles
Source: Front Nutr. 2025 Jul 3;12:1571990. doi: 10.3389/fnut.2025.1571990 (PMC12267017; doi:10.3389/fnut.2025.1571990)
Supplement: Supplementary file 2 [file Table_2.docx]

STROBE Statement—checklist of items that should be included in reports of observational studies

|  | Item No. | Recommendation | Page  No. | Relevant text from manuscript |
| --- | --- | --- | --- | --- |
| **Title and abstract** | 1 | (*a*) Indicate the study’s design with a commonly used term in the title or the abstract |  | Associations between nutritional status and cognitive impairment in older adults: Results from the NHANES 2011-2014 cycles |
|  |  | (*b*) Provide in the abstract an informative and balanced summary of what was done and what was found |  | Elevated CONUT scores were associated with an increased prevalence of cognitive impairment in older adults, indicating its potential utility in identifying individuals at risk of cognitive decline. |
| Introduction | | | |  |
| Background/rationale | 2 | Explain the scientific background and rationale for the investigation being reported |  | As the world's population ages, cognitive dysfunction has become more prevalent. As a growing public health problem, cognitive impairment now poses a significant challenge to health care systems. |
| Objectives | 3 | State specific objectives, including any prespecified hypotheses |  | Although several studies have supported the predictive and prognostic value of the CONUT score, the utility of the score in cognitively impaired populations has not been clarified. Therefore, the purpose of this study was to clarify the relationship between the CONUT score and cognitive function in an older population. |
| Methods | | | |  |
| Study design | 4 | Present key elements of study design early in the paper |  | This cross-sectional study utilized data from the National Health and Nutrition Examination Survey (NHANES) 2011-2014 cycles. |
| Setting | 5 | Describe the setting, locations, and relevant dates, including periods of recruitment, exposure, follow-up, and data collection |  | During the 2011-2014 cycles, NHANES implemented cognitive function assessments specifically targeting older adults aged 60-80 years. From 3472 participants who completed cognitive questionnaires across both cycles, we excluded those with missing CONUT scores. This study utilized data from the NHANES database, where trained professionals collected demographic characteristics, laboratory measurements, and questionnaire responses through standardized protocols. All data are publicly accessible via the NHANES website (https://wwwn.cdc.gov/nchs/nhanes/). |
| Participants | 6 | (*a*) *Cohort study*—Give the eligibility criteria, and the sources and methods of selection of participants. Describe methods of follow-up  *Case-control study*—Give the eligibility criteria, and the sources and methods of case ascertainment and control selection. Give the rationale for the choice of cases and controls  *Cross-sectional study*—Give the eligibility criteria, and the sources and methods of selection of participants |  | During the 2011-2014 cycles, NHANES implemented cognitive function assessments specifically targeting older adults aged 60-80 years. From 3472 participants who completed cognitive questionnaires across both cycles, we excluded those with missing CONUT scores. |
|  |  | (*b*) *Cohort study*—For matched studies, give matching criteria and number of exposed and unexposed  *Case-control study*—For matched studies, give matching criteria and the number of controls per case |  |  |
| Variables | 7 | Clearly define all outcomes, exposures, predictors, potential confounders, and effect modifiers. Give diagnostic criteria, if applicable |  | Three distinct methodologies were employed for the cognitive assessment of older adults aged 60 years or older within the NHANES.  This study employed the CONUT score to evaluate participants' nutritional status. The CONUT score was calculated based on serum total cholesterol levels, lymphocyte counts, and serum albumin concentrations (Supplementary Table 1). |
| Data sources/ measurement | 8* | For each variable of interest, give sources of data and details of methods of assessment (measurement). Describe comparability of assessment methods if there is more than one group |  | Demographic variables included age, gender, race, marital status, annual household income, and educational attainment. Laboratory analyses encompassed lymphocyte/neutrophil counts, total cholesterol (TC), and other biochemical parameters. Dietary nutrient intake – including energy, protein, total saturated fatty acid, carbohydrate, and fiber – was assessed through 24-hour dietary recall interviews. Cardiovascular disease (CVD) status was determined by self-reported physician diagnosis. |
| Bias | 9 | Describe any efforts to address potential sources of bias |  | We specifically extracted medications potentially influencing CONUT scores, including lipid-lowering agents, corticosteroids, and immunosuppressants.  The cutoff point for identifying cognitive impairment was determined by selecting the CERAD-WL, CERAD-DR, AFT, and DSST scores in the lowest quartile. This approach aligns with established methods in the literature for diagnosing cognitive impairment.  Given the limited number of participants classified as moderate or severe malnutrition (CONUT ≥5), we dichotomized participants into two groups for analytical purposes: normal (CONUT 0-1) and abnormal (CONUT 2-12). |
| Study size | 10 | Explain how the study size was arrived at |  | During the 2011-2014 cycles, NHANES implemented cognitive function assessments specifically targeting older adults aged 60-80 years. From 3472 participants who completed cognitive questionnaires across both cycles, we excluded those with missing CONUT scores. |

Continued on next page

| Quantitative variables | 11 | Explain how quantitative variables were handled in the analyses. If applicable, describe which groupings were chosen and why |  | Participants were stratified into normal nutrition (CONUT 0-1) and abnormal nutrition (CONUT 2-12) groups for baseline characteristic comparisons. Categorical variables were compared using Rao-Scott's χ² tests and reported as frequencies with weighted percentages, while continuous variables were analyzed via weighted t-tests and presented as weighted means ± standard deviations. |
| --- | --- | --- | --- | --- |
| Statistical methods | 12 | (*a*) Describe all statistical methods, including those used to control for confounding |  | The associations between nutritional status (both categorical groups and continuous CONUT scores) and cognitive function were evaluated using univariable and multivariable logistic regression models. Dose-response relationships between CONUT scores and cognitive impairment were visualized through restricted cubic spline (RCS) analysis with three knots. |
|  |  | (*b*) Describe any methods used to examine subgroups and interactions |  | Participants were stratified into normal nutrition (CONUT 0-1) and abnormal nutrition (CONUT 2-12) groups for baseline characteristic comparisons. |
|  |  | (*c*) Explain how missing data were addressed |  | Missing covariates with <20% missingness were retained in analyses, and missing values were imputed using multiple imputation by chained equations (MICE package in R). |
|  |  | (*d*) *Cohort study*—If applicable, explain how loss to follow-up was addressed  *Case-control study*—If applicable, explain how matching of cases and controls was addressed  *Cross-sectional study*—If applicable, describe analytical methods taking account of sampling strategy |  | All statistical analyses were conducted using R software (version 4.3.1) with appropriate weighting procedures through the "survey" package, in accordance with NHANES analytical guidelines. |
|  |  | (*e*) Describe any sensitivity analyses |  | The associations between nutritional status (both categorical groups and continuous CONUT scores) and cognitive function were evaluated using univariable and multivariable logistic regression models. |
| Results | | | | |
| Participants | 13* | (a) Report numbers of individuals at each stage of study—eg numbers potentially eligible, examined for eligibility, confirmed eligible, included in the study, completing follow-up, and analysed |  | The initial study cohort comprised 3,472 older adults who completed cognitive questionnaires. |
|  |  | (b) Give reasons for non-participation at each stage |  | After excluding 538 participants with incomplete cognitive assessments, 107 lacking lymphocyte count data, and 68 with missing albumin or TC measurements, the final analytical sample included 2,755 participants aged 60-80 years |
|  |  | (c) Consider use of a flow diagram |  | Figure 1 |
| Descriptive data | 14* | (a) Give characteristics of study participants (eg demographic, clinical, social) and information on exposures and potential confounders |  | Table 1 presents baseline characteristics stratified by nutritional status. |
|  |  | (b) Indicate number of participants with missing data for each variable of interest |  | Missing covariates with <20% missingness were retained in analyses, and missing values were imputed using multiple imputation by chained equations |
|  |  | (c) *Cohort study*—Summarise follow-up time (eg, average and total amount) |  | Not Applicable |
| Outcome data | 15* | *Cohort study*—Report numbers of outcome events or summary measures over time |  |  |
|  |  | *Case-control study—*Report numbers in each exposure category, or summary measures of exposure |  |  |
|  |  | *Cross-sectional study—*Report numbers of outcome events or summary measures |  | The CONUT score distribution histogram (Figure 2) revealed that 70.2% of participants maintained good nutritional control (scores 0-1), while 25.0% exhibited moderate malnutrition (scores 2-4), and 0.8% demonstrated severe malnutrition (scores ≥5). |
| Main results | 16 | (*a*) Give unadjusted estimates and, if applicable, confounder-adjusted estimates and their precision (eg, 95% confidence interval). Make clear which confounders were adjusted for and why they were included |  | In the crude model, elevated CONUT scores were linked to increased odds of cognitive dysfunction (OR: 1.345 [95% CI, 1.254–1.442]). Similarly, direct correlations were observed between CONUT scores and CERAD-WL, CERAD-DR, AFT, and DSST performance. Model 1 adjusted for age, BMI, and gender; Model 2 further incorporated comorbidities (diabetes mellitus, cardiovascular disease, hypertension); Model 3 additionally adjusted for medications influencing CONUT components. |
|  |  | (*b*) Report category boundaries when continuous variables were categorized |  | The CONUT score distribution histogram (Figure 2) revealed that 70.2% of participants maintained good nutritional control (scores 0-1), while 25.0% exhibited moderate malnutrition (scores 2-4), and 0.8% demonstrated severe malnutrition (scores ≥5). Table 1 presents baseline characteristics stratified by nutritional status. |
|  |  | (*c*) If relevant, consider translating estimates of relative risk into absolute risk for a meaningful time period |  | In the crude model, elevated CONUT scores were linked to increased odds of cognitive dysfunction (OR: 1.345 [95% CI, 1.254–1.442]). |

Continued on next page

| Other analyses | 17 | Report other analyses done—eg analyses of subgroups and interactions, and sensitivity analyses |  | RCS analyses revealed a significant L-shaped association between CONUT scores and cognitive impairment (Figure 3). |
| --- | --- | --- | --- | --- |
| Discussion | | | | |
| Key results | 18 | Summarise key results with reference to study objectives |  | Our NHANES-based study provides robust epidemiological evidence for the association between nutritional status (assessed via CONUT score) and cognitive dysfunction in older adults. The findings demonstrate that elevated CONUT scores maintain an independent and dose-dependent association with cognitive impairment, even after rigorous adjustment for clinical baseline characteristics and pharmacological confounders. |
| Limitations | 19 | Discuss limitations of the study, taking into account sources of potential bias or imprecision. Discuss both direction and magnitude of any potential bias |  | However, this study has several limitations. The cross-sectional design precludes definitive conclusions regarding the temporal sequence and causal relationship between CONUT scores and cognitive impairment. |
| Interpretation | 20 | Give a cautious overall interpretation of results considering objectives, limitations, multiplicity of analyses, results from similar studies, and other relevant evidence |  | This study found a greater prevalence of cognitive impairment among the participants in the group with nonnormal CONUT scores. Even after adjusting for potential confounders, cognitive impairment was independently associated with the CONUT score. |
| Generalisability | 21 | Discuss the generalisability (external validity) of the study results |  | Given the important role of malnutrition in cognitive impairment, this study's findings suggest that the CONUT score can help in the identification of elderly individuals with cognitive impairment. |
| Other information | |  | | |
| Funding | 22 | Give the source of funding and the role of the funders for the present study and, if applicable, for the original study on which the present article is based |  | This study was supported by a Grant from the Natural Science Foundation of Sichuan Province (Grant No. 24NSFSC2348). |

*Give information separately for cases and controls in case-control studies and, if applicable, for exposed and unexposed groups in cohort and cross-sectional studies.

**Note:** An Explanation and Elaboration article discusses each checklist item and gives methodological background and published examples of transparent reporting. The STROBE checklist is best used in conjunction with this article (freely available on the Web sites of PLoS Medicine at http://www.plosmedicine.org/, Annals of Internal Medicine at http://www.annals.org/, and Epidemiology at http://www.epidem.com/). Information on the STROBE Initiative is available at www.strobe-statement.org.
